# Data for the effects of rLj-RGD3 on normal tissues of rats and its location in HeyA8 cells

**DOI:** 10.1016/j.dib.2017.03.033

**Published:** 2017-03-23

**Authors:** Yuanyuan Zheng, Jianmei Han, Yuping Wang, Qi Jiang, Yue Wang, Li Lv, Rong Xiao, Jihong Wang

**Affiliations:** a School of Life Sciences, Liaoning Normal University, Dalian 116081, PR China; b Department of Pharmacology, Dalian Medical University, Dalian 116044, PR China

**Keywords:** rLj-RGD3, HeyA8 cells, Safe evaluation, Location

## Abstract

Lj-RGD3 which contains three Arg-Gly-Asp (RGD) motifs was identified from the buccal glands of *Lampetra japonica*. In the present data article, acute toxicity of recombinant Lj-RGD3 (rLj-RGD3) was performed in Sprague Dawley (SD) rats. Tissue observation data of these SD rats treated with normal saline (NS) or rLj-RGD3 were shown. Furthermore, confocal microscope data were also shown to observe the location of FITC-labeled rLj-RGD3 in the ovarian cancer cells (HeyA8 cells). This paper contains data related to research concurrently published in “rLj-RGD3 induces apoptosis via the mitochondrial-dependent pathway and inhibits adhesion, migration and invasion of human HeyA8 cells via FAK pathway” (Q. Jiang, Q. Li, J. Han, M. Gou, Y. Zheng, B. Li, R. Xiao, J. Wang, 2017) [1].

**Specifications Table**

**Table:** 

Subject area	*Biology, Biochemistry*
More specific subject area	*Safe evaluation of rLj-RGD3 and its location in HeyA8 cells*
Type of data	*Figures*
How data was acquired	*Tissue observation and confocal microscopy*
Data format	*Raw and analyzed*
Experimental factors	*NS or rLj-RGD3 was intravenously injected in SD rats; rLj-RGD3 was labeled with FITC and then added into HeyA8 cells.*
Experimental features	*NS or rLj-RGD3 was used to treat SD rats and their tissues were observed; rLj-RGD3 was labeled with FITC and then added into the HeyA8 cells. The location of rLj-RGD3 was observed by a confocal laser scanning microscope.*
Data source location	*Dalian, China*
Data accessibility	*Data are available with this article.*

**Value of the data**

• The data are valuable for the safety of rLj-RGD3 treatment in rats which were bearing tumors.
• The data are valuable for the acute toxicity of rLj-RGD3 in rats.
• The data are valuable for the location of rLj-RGD3 in the HeyA8 cells.

## 1 Data

**Fig. 1:**
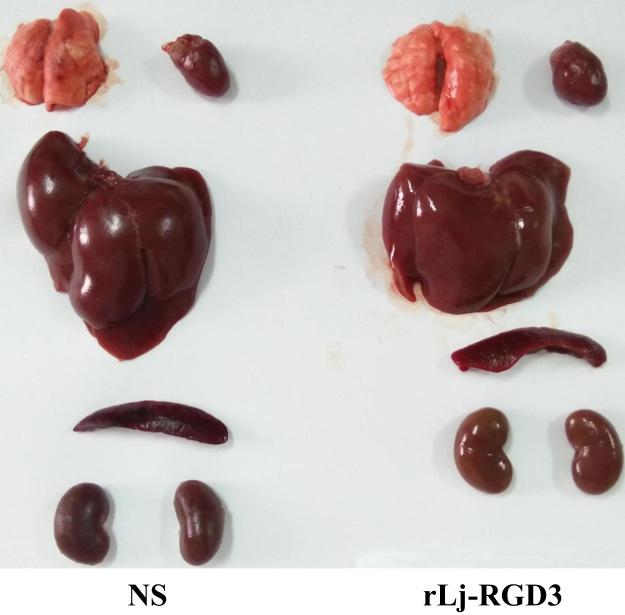
Tissues including lungs, hearts, livers, spleens and kidneys were shown after the SD rats were intravenously injected with NS and 20 mg/kg rLj-RGD3, respectively.

**Fig. 2:**
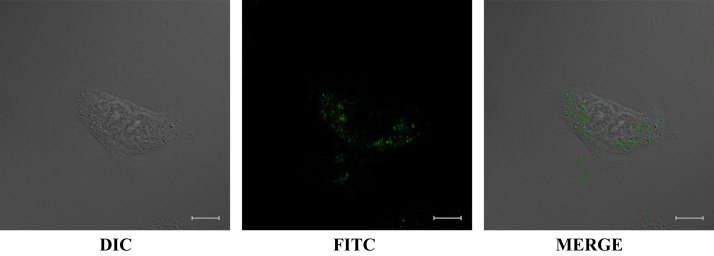
The location of FITC-labeled rLj-RGD3 in the HeyA8 cells.

The data of this article provide the tissue observation of Sprague Dawley (SD) rats which have been treated with normal saline (NS) or rLj-RGD3. Fig. 1 showed the lungs, hearts, livers, spleens and kidneys which were isolated from NS or rLj-RGD3 treated SD rats. In addition, confocal microscope data were shown to observe the location of FITC-labeled rLj-RGD3 in the HeyA8 cells (Fig. 2).

## 2 Experimental design, materials and methods

### 2.1 Safety evaluation of rLj-RGD3 treatment in SD rats

Acute toxicity of rLj-RGD3 was performed in Centre for Safety Evaluation and Research of Drugs, Institute of Laboratory Animal Science (ILAS), Chinese Academy of Medical Sciences (GLP11001029). 40 SD rats (20 males and 20 females) with the age of 6 weeks were random divided into two groups. Subsequently, these rats were intravenously injected with NS (negative control, 10 males and 10 females) and 20 mg/kg rLj-RGD3 (1000 times of clinical dosage of rLj-RGD3, 20 μg/kg, 10 males and 10 females), respectively. After 15 days, these mice were sacrificed and the tissues were observed.

### 2.2 Cell culture

DMEM (GIBCO, USA) containing 10% (vol/vol) fetal bovine serum (FBS, GIBCO) was used to culture HeyA8 cells in a humidified incubator with 5% CO_2_ at 37 °C [1].

### 2.3 Fluorescent labeling and staining

According to the manufacturer׳s instructions, the purified rLj-RGD3 was mixed with FITC (HOOK^™^ FITC Labeling Kit, G Biosciences) which is a commonly used reagent for labeling proteins. After removing the unconjugated dye, the labeled rLj-RGD3 (6 μM, final concentration) was added into the HeyA8 cells for 1 h. Subsequently, these HeyA8 cells were fixed with 4% paraformaldehyde for 10 min. Next, the HeyA8 cells were washed with PBS twice and observed by a laser scanning confocal microscope (Carl Zeiss, Germany) at 400× magnification.
