# Supplementary material for: Data for the effects of rLj-RGD3 on normal tissues of rats and its location in HeyA8 cells
Source: Data Brief. 2017 Mar 23;12:77–80. doi: 10.1016/j.dib.2017.03.033 (PMC5376250; doi:10.1016/j.dib.2017.03.033)
Supplement: Supplementary file 1 — Supplementary material [file mmc1.docx]

Conflict of interest form

Dear Editors:

The authors claim that we do not have any conflict of interest and none of the material in the paper has been published or is under consideration for publication elsewhere.

Yuanyuan Zheng

Jianmei Han

Yuping Wang

Qi Jiang

Yue Wang

Li Lv

Rong Xiao

Jihong Wang
